# Functional and Molecular Changes of the Bladder in Rats with Crushing Injury of Nerve Bundles from Major Pelvic Ganglion to the Bladder: Role of RhoA/Rho Kinase Pathway

**DOI:** 10.3390/ijms140917511

**Published:** 2013-08-27

**Authors:** Su Jin Kim, Dong Sup Lee, Woong Jin Bae, Seol Kim, Sung Hoo Hong, Ji Youl Lee, Tae-Kon Hwang, Sae Woong Kim

**Affiliations:** 1 Department of Urology, Seoul St. Mary’s Hospital, the Catholic University of Korea, 505 Banpo-dong, Seocho-gu, Seoul 137-701, Korea; E-Mails: hygeia@catholic.ac.kr (S.J.K.); bwoong@catholic.ac.kr (W.J.B.); godfafa@catholic.ac.kr (S.K.); toomey@catholic.ac.kr (S.H.H.); uroljy@catholic.ac.kr (J.Y.L.); tkhwang@catholic.ac.kr (T.-K.H.); 2 Department of Urology, St. Vincent’s Hospital, the Catholic University of Korea, 93 Jungbu-daero, Paldal-gu, Suwon 442-723, Gyeonggi-do, Korea; E-Mail: bbangbbangi@naver.com

**Keywords:** urinary bladder, receptor, muscarinic, Rho-associated kinase, autonomic ganglia, injury, nerve crush, rat

## Abstract

Voiding dysfunction is a common complication after radical pelvic surgery. To reduce this complication, nerve-sparing radical pelvic surgery was introduced. However, several patients experienced voiding difficulty despite nerve-sparing radical pelvic surgery. Thus, we investigated the functional and molecular changes of the bladder in rats, which demonstrated voiding dysfunction induced by nerve damage during nerve-sparing radical pelvic surgery. Male rats were used and assigned to normal, sham-operated, and bilateral crushing nerve bundles from major pelvic ganglion (MPG) to bladder group. After one, two, and four-week crushing injury, significantly decreased contractile response and increased connective tissue of the detrusor were observed and these results were reliable findings with voiding difficulty following nerve-sparing radical pelvic surgery. After crushing injury, significantly increased M2 muscarinic receptor expression was observed and this might be regarded as the compensatory response. However, M3 muscarinic receptor expression was not significantly changed. The expression of RhoA, ROCK-α, and ROCK-β was significantly increased after one, two, and four-week crushing injury. From these results, the down-regulation of RhoA/Rho kinase pathway might lead to the decreased bladder contractility after crushing injury of nerve bundles from MPG to the bladder despite of the compensated up-regulation of M2 muscarinic receptor.

## 1. Introduction

Prostate and rectal cancers are common pelvic organ malignancies in elderly men, and the prevalence of these cancers has increased. Currently, the survival rate of these cancers after treatment has improved because of improved treatment methods to eradicate these tumors [1], thus, more cancer survivors can now live much longer than in the past. Therefore, it is important to improve quality of life (QoL) in addition to eradicating cancers for these patients [2,3].

Voiding dysfunction is a common complication after surgical treatment of pelvic malignancy, and the symptoms can be as diverse as frequent urination, urinary incontinence, voiding by abdominal staining, and urinary retention. The majority of investigators who study QoL in patients after surgery of pelvic malignancy observed that voiding dysfunction was bothersome and disturbed the patients’ normal activities [4–7]. Among abnormal urination symptoms, voiding difficulty is a significant disability because the patient suffers from increased residual urine volume due to incomplete bladder emptying. In severe cases, some patients may experience urinary retention, and in this condition they can no longer urinate by themselves. Voiding by abdominal straining and urinary retention after surgery of pelvic malignancy are related to injury of peripheral nerves, such as the autonomic pelvic nerve. As the autonomic pelvic nerve that controls urination is located near the rectum and the prostate, injury of the autonomic pelvic nerve can occur during surgery [8,9]. Difficulty in bladder emptying is caused by impaired bladder contractility that is associated with detrusor underactivity and diminished bladder sensation after surgery. Iatrogenic nerve injury-induced detrusor underactivity is common condition; however, research on iatrogenic nerve injury-induced detrusor underactivity is lacking compared with research on other voiding dysfunctions [10]. Bladder contraction can be regulated by the M2 and M3 muscarinic receptors or RhoA/Rho kinase pathways existing in the bladder. Therefore, voiding dysfunction such as detrusor underactivity is induced by change in the activities of M2 and M3 muscarinic receptors or RhoA/Rho kinase pathway in the bladder [11].

Because the number of survivors who suffer from voiding problem has increased, several recent attempts have been made to reduce voiding complications, such as nerve-sparing radical surgery. Nonetheless, some patients have experienced voiding dysfunctions, such as incomplete bladder emptying and urinary retention, in spite of the nerve-sparing radical pelvic surgery. Therefore, it is necessary to understand the physiology of voiding dysfunction after nerve-sparing radical pelvic surgery using representative animal models. However, only a few studies on voiding dysfunction after nerve-sparing radical pelvic surgery have been performed. Therefore, we studied the changes of the bladder in animals with voiding dysfunction after injury, which represents nerve damage during nerve-sparing radical pelvic surgery. Our study also investigated the contribution of M2 and M3 muscarinic receptors and RhoA/Rho kinase pathway to bladder contraction after injury.

## 2. Results

### 2.1. Bladder Weights in the Control, Sham-Operated, and Crushing Injury on Nerve Bundles from the Major Pelvic Ganglion (MPG) to the Bladder Group at One, Two, and Four Weeks after Injury

**Figure 1:**
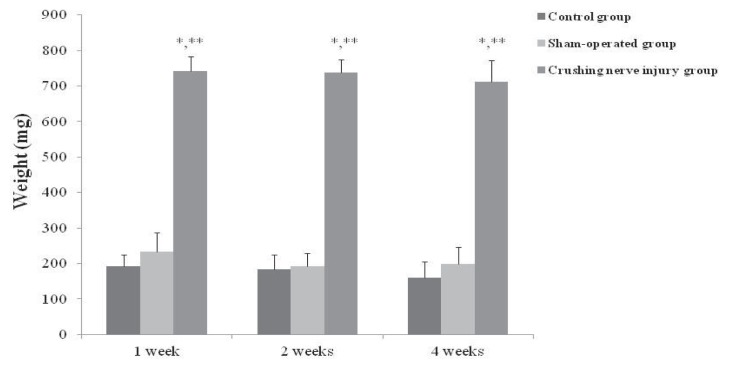
Bladder weights in the control, sham-operated, and crushing injury on nerve bundles from the MPG to the bladder groups at one, two, and four weeks after injury. Measurement of bladder weights was done in control (*n* = 5), sham-operated (*n* = 5), and crushing injury on nerve bundles from MPG (*n* = 10) groups at each of one, two, and four weeks after injury. Results are expressed as mean ± standard deviation of the mean. * *p* < 0.05 compared with the control group, ** *p* < 0.05 compared with the sham-operated group.

At one week after injury, the bladder weight of crushing injury on nerve bundles from the MPG group (740.9 ± 31.6 mg) was significantly increased compared with the control and sham-operated groups (193.4 ± 31.7 and 232.8 ± 55.2 mg, respectively) (*p* < 0.05). The bladder weight of crushing injury on nerve bundles from the MPG group (738.2 ± 36.2 mg) was significantly increased compared with the control and sham-operated groups (183.2 ± 42.5 and 193.4 ± 35.2 mg, respectively) at two weeks after injury (*p* < 0.05). Similar with one and two weeks after injury, the bladder weight of crushing injury on nerve bundles from the MPG group (711.5 ± 60.4 mg) was significantly increased compared with the control and sham-operated groups (160.9 ± 44.8 and 197.8 ± 48.2 mg, respectively) (*p* < 0.05) (Figure 1).

### 2.2. Contractile Response of the Bladder Strip in the Control, Sham-Operated, and Crushing Injury on Nerve Bundles from the MPG to the Bladder Group at One, Two, and Four Weeks after Injury

**Figure 2:**
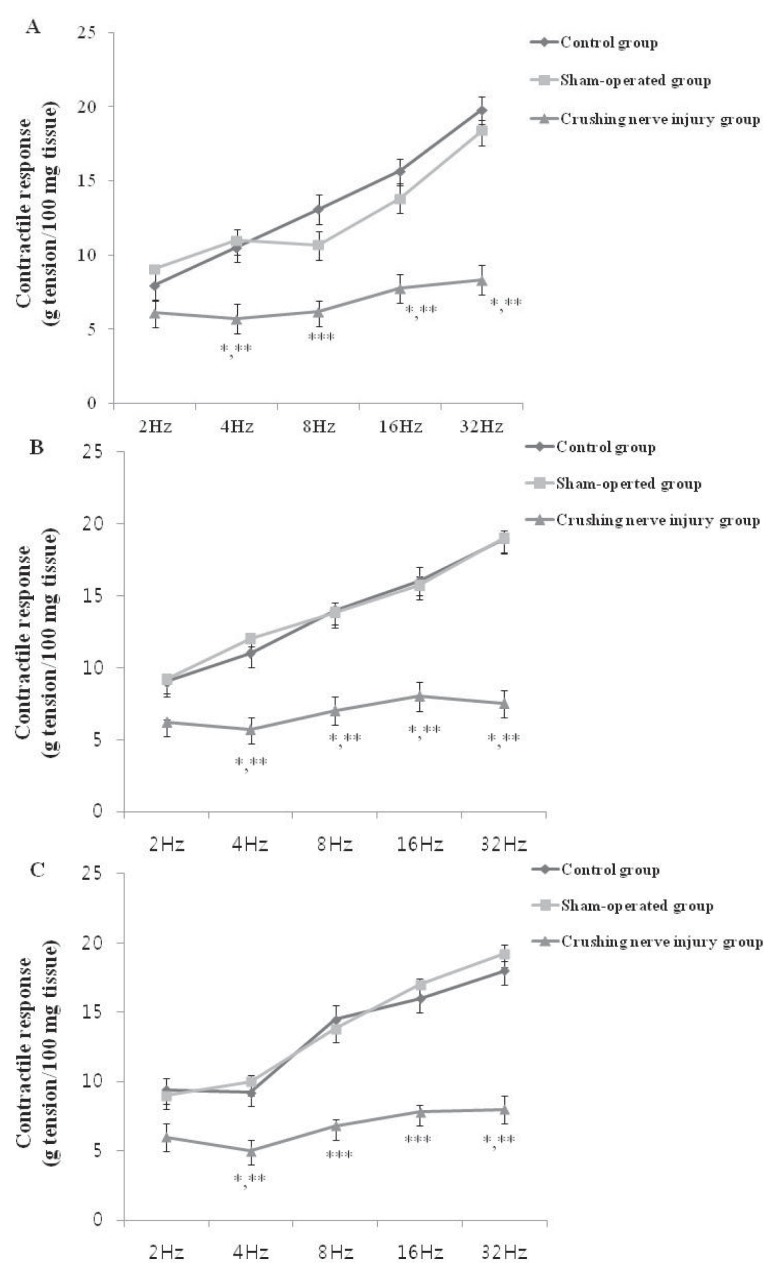
Contractile response to electrical field stimulation in the control, sham-operated, and crushing injury on nerve bundles from the MPG to the bladder group at one (**A**); two (**B**); and four (**C**) weeks after injury. The number of rats analyzed at each of one, two, and four weeks after injury was five in the control group, five in the sham-operated group, and 10 in the crushing injury on nerve bundles from MPG group. Results are expressed as mean ± standard deviation of the mean. * *p* < 0.05 compared with the control group, ** *p* < 0.05 compared with the sham-operated group, *** *p* < 0.01 compared with the control and sham-operated groups.

The results of EFS of bladder strips were shown in Figure 2. At one week after crushing nerve injury, the contractile response was significantly decreased compared with the control and sham-operated groups at each of the four frequencies (2, 4, 8, and 32 Hz) (*p* < 0.05) (Figure 2A). In addition, the significantly decrease contractile response at two and four weeks after injury was observed compared with the control and sham-operated groups (Figure 2B,C).

### 2.3. The Smooth Muscle to Collagen Ratio of the Detrusor in the Control, Sham-Operated, and Crushing Injury on Nerve Bundles from the MPG to the Bladder Group at One, Two, and Four Weeks after Injury

**Figure 3:**
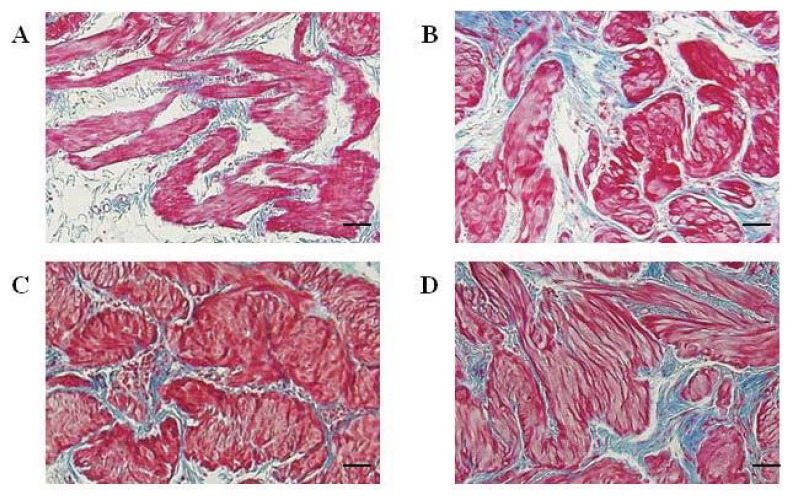

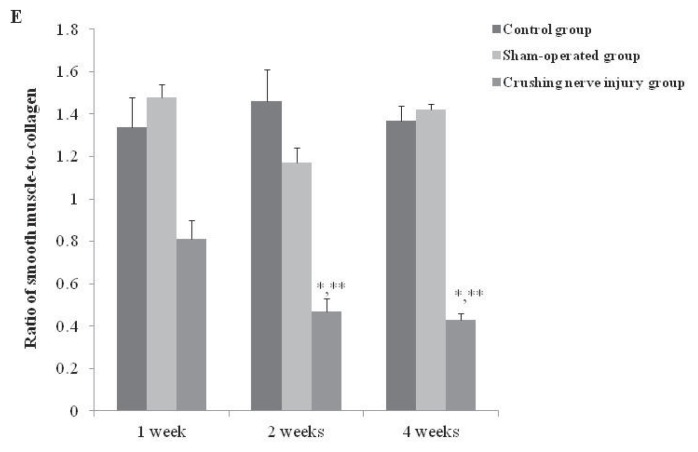
Distribution of smooth muscle and collagen in detrusor tissue from the bladder in the control and crushing injury on nerve bundles from the MPG to the bladder groups at one, two, and four weeks after injury; Smooth muscle and collagen in detrusor from the bladder of the control (**A**) and crushing injury on nerve bundles from MPG to the bladder (**B**) groups at one week after injury; Smooth muscle and collagen in detrusor from the bladder of the crushing injury on nerve bundles from MPG to the bladder groups at two (**C**) and four (**D**) weeks after injury; (**E**) The smooth muscle to collagen ratio in the control, sham-operated, and crushing injury on nerve bundles from the MPG to the bladder group at one, two, and four weeks after injury. The number of rats analyzed at each of one, two, and four weeks after injury was five in the control group, five in the sham-operated group, and 10 in the crushing injury on nerve bundles from MPG group. Results are expressed as mean ± standard deviation of the mean. * *p* < 0.05 compared with the control group, ** *p* < 0.05 compared with the sham-operated group. Bar scale = 500 μm.

After one week after crushing nerve injury, the hypertrophy of smooth muscle and increased deposition of collagen were observed compared with the control group (Figure 3A,B). More hypertrophied smooth muscle and significantly increased expression of collagen were observed at two and four weeks after injury (Figure 3C,D). The smooth muscle to collagen ratio of the detrusor in rats with crushing nerve injury (0.81 ± 0.09) was decreased compared with the control (1.34 ± 0.14) and sham-operated (1.48 ± 0.06) groups but there was no significance at one week after injury. In addition, significantly decreased smooth muscle to collagen ratio of detrusor was observed at two (0.47 ± 0.06) and four (0.43 ± 0.03) weeks after injury compared with the control and sham-operated groups (*p* < 0.05) (Figure 3E).

### 2.4. Analysis of the Expression of M2 and M3 Muscarinic Receptors in the Bladder from the Control, Sham-Operated, and Crushing Injury on Nerve Bundles from the MPG to the Bladder Group at One, Two, and Four Weeks after Injury

**Figure 4:**
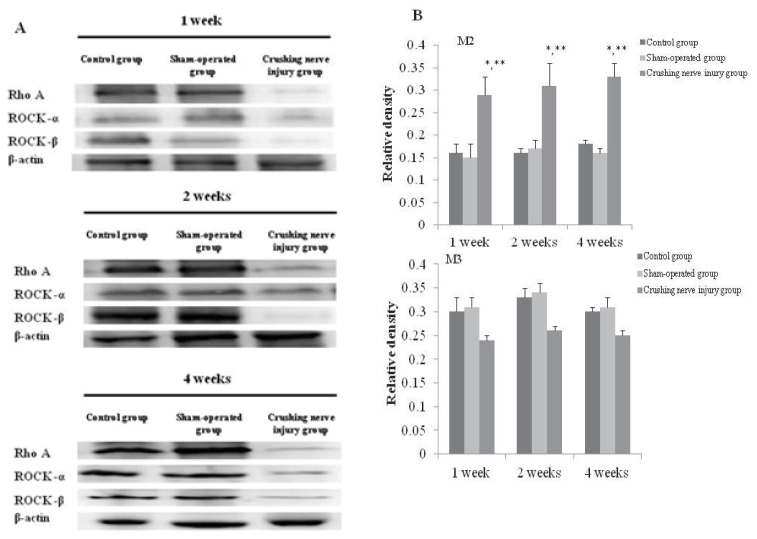
Expressions of M2 and M3 muscarinic receptors in the bladder from the control, sham-operated, and crushing injury on nerve bundles from the MPG to the bladder group at one, two, and four weeks after injury (**A**); Relative protein expression of M2 and M3 muscarinic receptors of the bladder. The number of rats analyzed at each of one, two, and four weeks after injury was five in the control group, five in the sham-operated group, and 10 in the crushing injury on nerve bundles from MPG group (**B**). Results are expressed as mean ± standard deviation of the mean. * *p* < 0.05 compared with the control group, ** *p* < 0.05 compared with the sham-operated group.

After one-week crushing injury, the significantly increased expression of M2 muscarinic receptor was observed compared with the control and sham-operated group (*p* < 0.05). Significantly increased expression of M2 muscarinic receptor was maintained at two and four weeks after crushing injury (*p* < 0.05) (Figure 4). The expression of M3 muscarinic receptor was decreased at one week after crushing injury; however, there was no significant difference compared with the control and sham-operated group. After two- and four-week crushing injury, the expression of M2 muscarinic receptor was decreased; however, there was no significant change compared with the control and sham-operated group (Figure 4).

### 2.5. Analysis of the Expression of RhoA, ROCK-α, and ROCK-β in the Bladder from the Control, Sham-Operated, and Crushing Injury on Nerve Bundles from the MPG to the Bladder Group at One, Two, and Four Weeks after Injury

**Figure 5:**
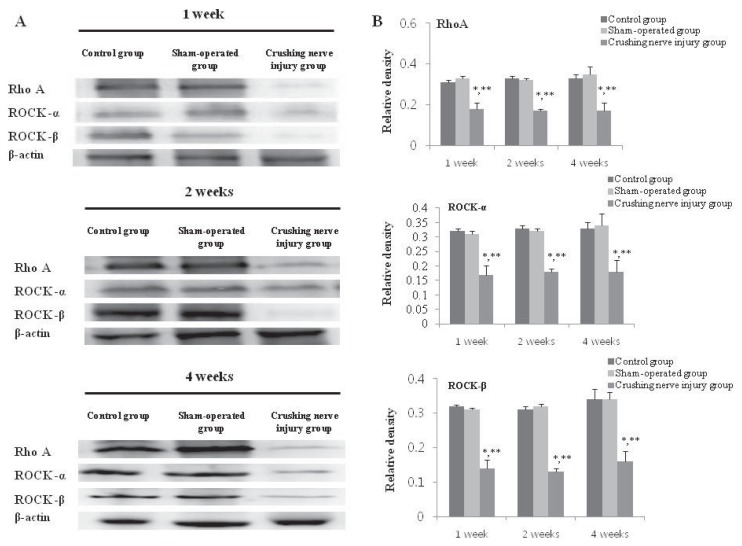
Expressions of RhoA, ROCK-α, and ROCK-β in the bladder from the control, sham-operated, and crushing injury on nerve bundles from the MPG to the bladder group at one, two, and four weeks after injury (**A**); Relative protein expression of RhoA, ROCK-α, and ROCK-β of the bladder. The number of rats analyzed at each of one, two, and four weeks after injury was five in the control group, five in the sham-operated group, and 10 in the crushing injury on nerve bundles from MPG group (**B**). Results are expressed as mean ± standard deviation of the mean. * *p* < 0.05 compared with the control group, ** *p* < 0.05 compared with the sham-operated group.

After one-week crushing injury, the significantly increased expression of RhoA, ROCK-α, and ROCK-β was observed compared with the control and sham-operated group (*p* < 0.05). Significantly increased expression of RhoA, ROCK-α, and ROCK-β was maintained at two and four weeks after crushing injury (*p* < 0.05) (Figure 5).

## 3. Discussion

In this study, significantly increased bladder weights of the rats after crushing nerve injury was noted after one week, and this increase was maintained at two- and four-week after injury compared with normal rats. Functionally, bladder contractility was significantly decreased at one week after injury, and the decreased bladder contractility continued through four weeks. These results indicated that crushing injury on nerve bundles from the MPG to the bladder induced detrusor underactivity. In addition, we observed the histologic changes such as hypertrophied smooth muscle and significant decrease of the smooth muscle-to-collagen ratio and these results were also supportive findings for the development of detrusor underactivity after crushing nerve injury. Moreover, we demonstrated the changes of RhoA, ROCK-α, and ROCK-β as well as M2 and M3 muscarinic receptors in the bladder after crushing nerve injuries.

Previously, some investigators studied functional bladder changes in animals after transecting autonomic pelvic nerves [12]; however, nerve transaction-induced voiding dysfunction is not relevant to the voiding dysfunction after radical pelvic surgery with the nerve-sparing technique. In the present study, we performed bilateral crushing injury on nerve bundles from the MPG to the bladder to represent voiding dysfunction after nerve-sparing radical pelvic surgery. The nerve crushing method was adopted from the studies to make animal models represent erectile dysfunction, induced by nerve-sparing radical pelvic surgery in rats [13,14].

Impaired bladder contractility was not improved until four weeks after bilateral crushing injury on nerve bundles from MPG, and this outcome might demonstrate that this model is acceptable for use as a model for nerve-sparing radical pelvic surgery-induced voiding dysfunction. In the present study, bladder weight after crushing injury was increased one week after injury, and this finding supported decreased bladder contractility. Guven *et al.* [15] also observed that increased bladder weight was related to decreased bladder contractility. Those authors found not only a time-dependent decrease in bladder contractility but also a coincidence of time between decreased bladder contractility and increased bladder weight after bladder outlet obstruction (BOO) in rabbits. Similar with the results of Guven *et al.* [15], significantly decreased bladder contractility was observed as bladder weight was significantly increased after injury. In addition, we observed similar morphologic changes of bladder wall. Smooth muscle-to-collagen ratio was significantly decreased and increased collagen infiltration was noted after injury.

The role of M2 and M3 muscarinic receptor subtypes on bladder contraction has been widely studied in various animal models. In addition, RhoA/Rho kinase pathway is important for smooth muscle contraction in tissues such as the intestine and the bladder. Therefore, we compared alterations in M2 and M3 muscarinic receptor subtypes and RhoA, ROCK-α, and ROCK-β by Western blot analysis. According to a study on the relationship among M2 and M3 muscarinic receptors and ROK using normal human detrusor smooth muscle, the M3 rather than the M2 muscarinic receptor played the predominant role in the detrusor smooth muscle by modulation of RhoA/Rho kinase and protein kinase C pathways, which are involved in Ca^2+^ sensitization [16]. These results suggested that M3 muscarinic receptors are more influential in normal bladder contraction. Therefore, most of the studies were performed about the roles and changes of M3 muscarinic receptors in various types of voiding dysfunction, such as overactive bladder (OAB) [17,18]. However, the present study demonstrated that M3 muscarinic receptor expression after crushing nerve injury was not significantly changed compared with the control. Furthermore, M2 muscarinic receptor expression, after crushing nerve injury, was significantly increased. From this result, we conclude that the M2 muscarinic receptors plays a more important role than the M3 muscarinic receptors after crushing nerve injury, and the role of the M2 or M3 receptor after nerve injury could be different from that of a normal bladder. Consistent with the results from the present study, several studies also have demonstrated that the M2 muscarinic receptors showed the more important role in denervated or hypertrophied bladder contraction [19,20]. Similar with the previous results, M2 muscarinic receptor expression after crushing injury was significantly increased compared with control or sham-operated groups, and this increased expression might be because of compensatory action to maintain bladder contraction.

However, bladder contractility was significantly decreased after crushing nerve injury despite the compensatory increased M2 receptor expression. From this finding, we suggest that the impaired bladder contractility was related to down-regulation of RhoA/Rho kinase pathway after crushing nerve injury, and similar results were found in a study to evaluate denervated bladder contractility after MPG electrocauterization [21]. The role of RhoA/Rho kinase to control bladder contraction has been demonstrated, as well as protein kinase C pathway. Moreover, RhoA/Rho kinase-mediated bladder contraction was involved with M2 muscarinic receptors rather than M3 muscarinic receptors [11]. From the significantly decreased expression of RhoA, ROCK-α, and ROCK-β in the present study, we suppose that impaired bladder contractility after crushing nerve injury may be induced by down-regulation of RhoA/Rho kinase pathway in spite of the compensatory increase expression of M2 muscarinic receptors. Moreover, our results may support the inconsistent efficacy of cholinergic medicine to treat incomplete emptying in clinical practice. Several patients suffered from persistent, incomplete emptying after radical pelvic surgery, despite taking cholinergic medicine [22,23]. Thus, RhoA/Rho kinase pathway may play a more important role in regulating voiding dysfunction induced by radical pelvic surgery than muscarinic receptors.

In this study, we investigated the changes of muscarinic receptors and RhoA/Rho kinase pathway associated with classic autonomic transmitter as acethylcholine. Although cholinergic neurotransmitter plays a major role to control voiding, there have been several reports about the potential action of non-adrenergic, non-cholinergic neurotransmitters [24]. Among them, there were several studies about the role of ATP, which showed activity after binding to purinergic receptors in the bladder. Purinergic signaling mediated by ATP is regarded to play a role in bladder contraction and relaxation. Previously, a majority of the studies demonstrated the role of purinergic signaling in detrusor overactivity observed in OAB or neurogenic bladder [25]. However, there were few studies about the influence of purinergic signaling on detrusor underactivity. Therefore, further studies about signaling pathway, mediated by non-adrenergic, non-cholinergic neurotransmitters such as ATP, are necessary to reveal the underlying mechanism of detrusor underactivity after nerve-sparing radical pelvis surgery.

## 4. Experimental Section

### 4.1. Animals

White male Sprague-Dawley rats aged 8 weeks with weight distribution of 250–300 gm (*n* = 60) were used in this study. The rats were divided into 3 groups: the control group (*n* = 15), the sham-operated group (*n* = 15), crushing injury on nerve bundles from the major pelvis ganglion (MPG) to the bladder group (*n* = 30). The experimental protocol was approved by the Catholic University Animal Ethics Committee (CUMC-2012-0049-02).

### 4.2. Surgical Procedures

**Figure 6:**
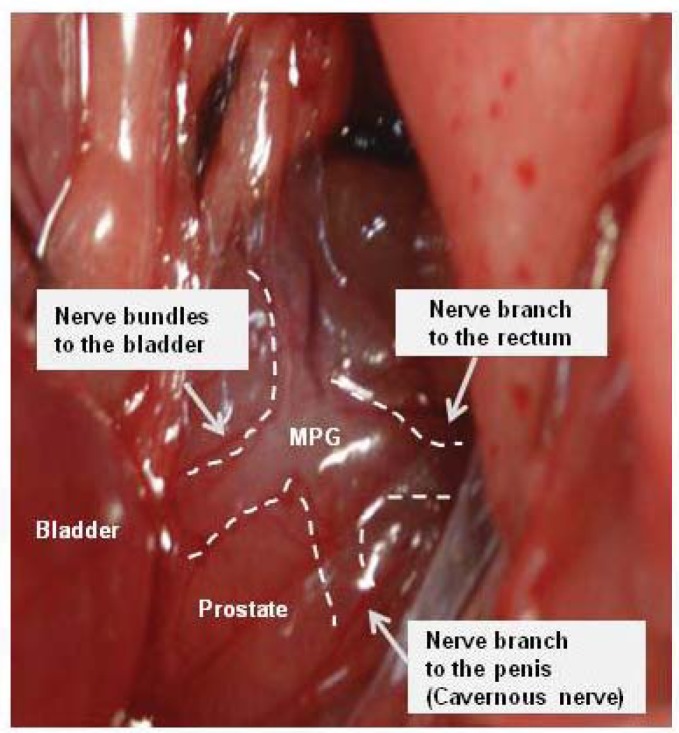
Anatomic appearance of nerve bundles from major pelvic ganglion (MPG) to the bladder. The nerve bundles were isolated and a crush injury induced using a hemostat clamp for 2 min in the crushing injury on nerve bundles from the MPG to the bladder group.

Tiletamine (Zoletil) 0.2 mL was injected intraperitoneally to anesthetize the animals. A lower midline incision was made and the prostate gland was exposed. After identifying of MPG on the lateral side to bilateral prostates [20], the nerve bundles from the MPG to the bladder were identified and isolated (Figure 6). In sham-operated group, there was no further surgical manipulation. In the crushing injury on nerve bundles from the MPG to the bladder group, the nerve bundles were isolated and a crush injury induced using a hemostat clamp for 2 min and the abdomen was closed. Functional and molecular evaluation of the bladder were performed in control, sham-operated, and crushing injury on nerve bundles from the MPG group after 1, 2, and 4 weeks.

### 4.3. *In Vitro* Investigation on the Contractile Response of Bladder Strip

**Figure 7:**
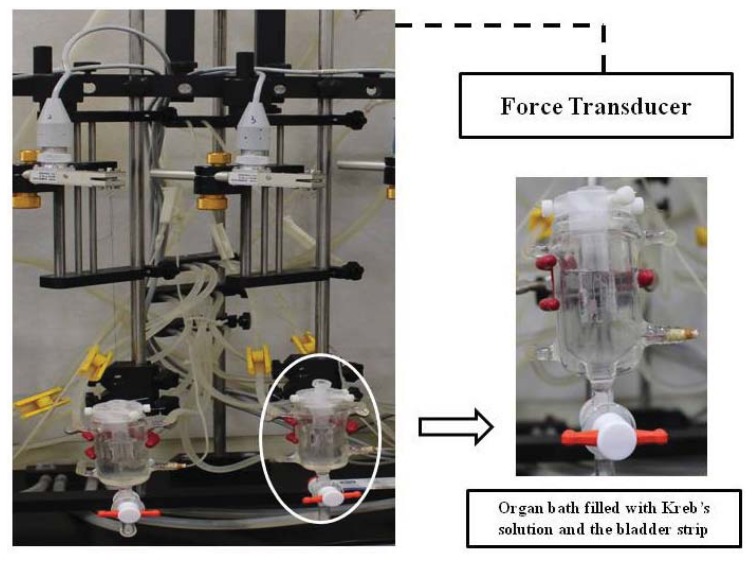
Isometric force recording to the electrical stimulation using bladder strip.

After 1, 2, and 4 weeks, the bladders were promptly removed by opening the lower abdomen and the bladders were placed in Krebs solution. Four strips were taken from the bladders of each of the 60 rats from the control, sham-operated, and crushing injury on nerve bundles from the MPG groups. After bladder weights were measured, the bladder body was longitudinally cut on the ventral side into 3 × 10 mm strips and placed into a 30-mL organ bath that was filled with Kreb’s solution and then connected to a force transducer (Grass model S48K, grass Instrument Division, Astro-Med Inc., West Warwick, RI, USA) (Figure 7). Tissues were allowed 1 h for establishment of equilibrium under a tension of 1 g at a temperature of 37 ± 1 °C and with continuous ventilation with 95% O_2_ and 5% CO_2_. Maximal contractile responses were recorded under electrical field stimulation at 2, 4, 8, 16, and 32 Hz for 30 s, each with pulses, 1 min in duration, at 80 V. The interval between stimulation was 10 min in the organ bath. The contractile responses were expressed in terms of the tension (gram), and the data are normalized to the tension generated with 100 mg of bladder tissue.

### 4.4. Investigation of Smooth Muscle and Collagen Component in the Detrusor

Bladder wall specimens were immediately fixed overnight in 10% formalin, washed, and stored in 70% alcohol at 4 °C until processed for paraffin-embedded tissue sectioning (5 μm). The bladder tissue was obtained for the Masson’s trichrome staining. After staining, the color distribution of the smooth muscle tissue was approximated by using the Adobe Photoshop CS 8.0. (Adobe System Inc., San Jose, CA, USA) After the entire color distribution of the image was calculated, we selected the muscle tissue distribution, expressed as the color red. In each section, at least 5 fields were selected at random on the muscle layer by coordinate mapping (×200 magnification). The ratio of smooth muscle-to-connective tissue was obtained by dividing the sum of smooth muscle area by the sum of connective tissue area of fields examined.

### 4.5. Western Blot Analyses for Muscarinic Receptors RhoA, ROCK-α, and ROCK-β Expression in Bladder Wall

Protein was homogenized in ice-cold lysis buffer containing 20 mM Tris-HCl, pH 8.0, 150 mM NaCl, 1 mM EDTA, 1% Triton X-100, 10 M leupeptin, 20 g/mL chymostatin, and 2 mM PMSF. Following centrifugation at 12,000× *g* for 30 min at 4 °C, the supernatant was extracted and quantified using the bicinchoninic acid (BCA) protein assay kit (Thermo Scientific, Rockford, IL, USA) The quantitated proteins (20 μg) were boiled in a loading buffer (62.6 mM Tris-HCl pH 6.8, 2% sodium dodecyl sulfate, 0.01% bromophenol blue, 10% glycerol, and 100 mM DTT). After being transferred onto Hybond-ECL nitrocellulose membrane (Amersham Biosciences, Freiburg, Germany), the membrane was blocked with 5% non-fat skim milk in Tris-buffered saline containing 0.1% Tween 20 and then incubated with antibody against Rho (1:2000; abcom, Cambridge, MA, USA), M2 (1:1000; abcam, Cambridge, MA, USA), M3 (1:500; abcam, , Cambridge, MA, USA), anti-ROCK-I/ROKβ antibody (1:250; BD Biosciences, San Jose, CA, USA), an anti-ROCK-II/ROKα antibody (1:1000; BD Biosciences), or β-actin, followed by incubation with the corresponding secondary antibody (Santa Cruz Biotechnology, CA, USA) conjugated to horseradish peroxidase. The ECL method (Amersham, Arlington Heights, IL, USA) was used for development of protein bands. The reaction was Luminescent Image Analysis System (LAS-3000; FUJIFILM, Tokyo, Japan) Densitometric analysis were performed using the Multi Gauge 3.0 software (FUJI Photo Film, Tokyo, Japan).

### 4.6. Statistic Analysis

The statistical analysis was done using SPSS 15.0 (SPSS Inc., Chicago, IL, USA). The data was expressed as the means ± standard deviations. The data for each group were compared using a one-way ANOVA and Bonferroni *post hoc* test. The significance was set at *p* < 0.05.

## 5. Conclusions

The present study demonstrated that crushing injury of nerve bundles form MPG to bladder led to the impaired bladder contractility and this might be represented to the voiding dysfunction such as detrusor underactivity following nerve-sparing radical pelvic surgery. In addition, RhoA/Rho kinase pathway may have a more important role to modulate bladder contractility than muscarinic receptors after crushing nerve injury. From these findings, the regulation of RhoA/Rho kinase pathway may be one of the therapeutic targets to improve bladder contractility in patients suffering voiding problems following nerve-sparing radical pelvic surgery.
